# Computerized diagnostic decision support systems—Isabel Pro versus ChatGPT-4 part II

**DOI:** 10.1093/jamiaopen/ooaf048

**Published:** 2025-06-16

**Authors:** Joe M Bridges, Xiaoqian Jiang, Michael Ige, Oluwatoniloba Toyobo

**Affiliations:** D. Bradley McWilliams School of Biomedical Informatics, University of Texas Health Science Center at Houston, Houston, TX 77030, United States; D. Bradley McWilliams School of Biomedical Informatics, University of Texas Health Science Center at Houston, Houston, TX 77030, United States; D. Bradley McWilliams School of Biomedical Informatics, University of Texas Health Science Center at Houston, Houston, TX 77030, United States; D. Bradley McWilliams School of Biomedical Informatics, University of Texas Health Science Center at Houston, Houston, TX 77030, United States

**Keywords:** artificial intelligence, diagnosis, computer assisted, Isabel Pro, ChatGPT-4

## Abstract

**Objective:**

Does a Tree-of-Thought prompt and reconsideration of Isabel Pro’s differential improve ChatGPT-4’s accuracy; does increasing expert panel size improve ChatGPT-4’s accuracy; does ChatGPT-4 produce consistent outputs in sequential requests; what is the frequency of fabricated references?

**Materials and Methods:**

Isabel Pro, a computerized diagnostic decision support system, and ChatGPT-4, a large language model. Using 201 cases from the New England Journal of Medicine, each system produced a differential diagnosis ranked by likelihood. Statistics were Mean Reciprocal Rank, Recall at Rank, Average Rank, Number of Correct Diagnoses, and Rank Improvement. For reproducibility, the study compared the initial expert panel run to each subsequent run, using the r-squared calculation from a scatter plot of each run.

**Results:**

ChatGPT-4 improved MRR and Recall at 10 to 0.72 but produced fewer correct diagnoses and lower average rank. Reconsideration of the Isabel Pro differential produced an improvement in Recall at 10 of 11%. The expert panel size of two produced the best result. The reproducibility runs were within 4% on average for Recall at 10, but the scatterplots showed an r-squared ranging from 0.44 to 034, suggesting poor reproducibility. Reference accuracy was 34.8% for citations and 37.8% for DOIs.

**Discussion:**

ChatGPT-4 performs well with images and electrocardiography and in administrative practice management, but diagnosis has not proven as promising.

**Conclusions:**

As noted above, the results demonstrate concerns for diagnostic accuracy, reproducibility, and reference citation accuracy. Until these issues are resolved, clinical usage for diagnosis will be minimal, if at all.

## Introduction

Usage of Large Language Models such as ChatGPT-4 has grown exponentially, creating an appetite for applications in medical specialties. Diagnostic decision support is an area where validation of the performance of a system relying on a non-specialist dataset, Common Crawl, might show greater accuracy than a specialized system such as Isabel Pro. Despite the enthusiasm for Generative Pre-Trained Large Language Models, such as ChatGPT-4, their use for clinical diagnosis remains cautious, if not minimal.^1^^,^^2^ Artificial intelligence shows great promise for many medical tasks—clinical notes, insurance requests, image analysis^3^^,^^4^—but diagnosis presents the challenge of patient harm if incorrect.^5^ The hesitation of clinicians to use these systems for diagnosis derives from the fabrication of references that underpin the presented diagnosis^6^ and the accuracy of the diagnosis.^7^ Fabricated references can easily lead a busy clinician to pursue an incorrect disease condition.^6^ Studies estimate that almost 800 000 US patients annually suffer permanent disability or death from misdiagnosis of dangerous diseases.^5^ Isabel Pro was selected as the best system for introduction into the primary care and outpatient clinics as the highest in diagnostic retrieval accuracy, fastest in speed of retrieval, most comprehensive differential diagnosis listing, reduced the number of incorrect diagnoses, and was most frequently used when available to clinicians.^8^ ChatGPT-4 was chosen after the authors introduced several cases into ChatGPT-4, Claude, and Bard, but finding that Claude and Bard were barred from producing a patient’s medical diagnosis. A recent study using a large language model specifically for diagnosis showed poorer results than the authors’ earlier studies.^9^ Following the earlier study by one of the authors,^10^ this study seeks to modify the ChatGPT-4 prompts to return a correct diagnosis within the top ten 95% of the time (Recall@10 = 0.95). Introduction of Tree of Thoughts prompts allows the LLM to progress in the decision process from simple decision-making to complex considerations and reconsiderations of reasoning trajectories.^11^ This study utilized the 201 cases from the earlier study, 36 cases from a study led by Dr Charles Friedman^12^ and 165 from the New England Journal of Medicine (chosen sequentially as published, but with a variety of patient demographics, disease conditions, and medical specialties), each with a clinical consensus diagnosis.^10^  Appendix S1 shows the patient demographics, sex at birth, and medical specialty by age range.

This study seeks answers to the following four research questions:

1. Does diagnostic accuracy improve when prompted to reconsider and re-rank the Isabel Pro differential?^5^ Does adding omitted diagnoses enhance diagnostic accuracy?
2. What is the effect of a Tree-of-Thought prompt (imagining a multi-expert panel considering the patient’s presentation) on the diagnostic accuracy of ChatGPT-4?^11^ What is the effect of panel size on diagnostic accuracy?^13^
3. Are the results of Tree-of-Thought prompts to ChatGPT-4 reproducible in sequential requests?
4. What is the accuracy of the reference citations produced by ChatGPT-4 using a Tree-of-Thought prompt?^6^

## Methods

### Software systems

This study compares the diagnostic performance of two systems, one relying on a highly curated reference dataset, Isabel Pro, and the other relying on a broad compilation of publicly available data of all manner, ChatGPT-4 and the Common Crawl. Clinical diagnosis is a complex task with implications for patient harm if incorrect, so accuracy, reproducibility, and basis for ranking diagnostic alternatives are essential for clinical usage.

#### Isabel Pro

Isabel Healthcare Ltd produces medical diagnosis decision support systems. One product is Isabel Pro, a CDDSS that employs a search algorithm to produce a differential diagnosis listing in order of the frequency with which the symptoms for a particular disease condition appear in a proprietary medical reference dataset. Isabel Pro’s reference dataset is updated monthly.

#### ChatGPT-4

ChatGPT-4 is a Generative Pre-trained Transformer (GPT) developed by OpenAI. ChatGPT-4 relies for training on Common Crawl, a publicly available dataset and one of the most extensive text datasets. Common Crawl contains medical articles and websites but does not contain highly respected medical reference texts. Common Crawl juxtaposes internet articles from predatory publishers alongside more reliable sources such as the Mayo Clinic website.

### Datasets

This study utilized the 201 cases from the earlier study, each with a clinical consensus diagnosis.^10^ The Study Dataset Demographics (see Appendix S1) show a male/female ratio of 1.25, 26% age 50-64, 20% 65-over, 18% 30-39, 13% 40-49, and 12% 17-29. Medical specialties comprising 65% of the cases are Infectious Diseases, Neoplasms, Rheumatology, Respiratory, Cardiovascular, Hematology, Endocrinology, and Gastrointestinal.

## Research questions and methodological approach

**Figure 1.**
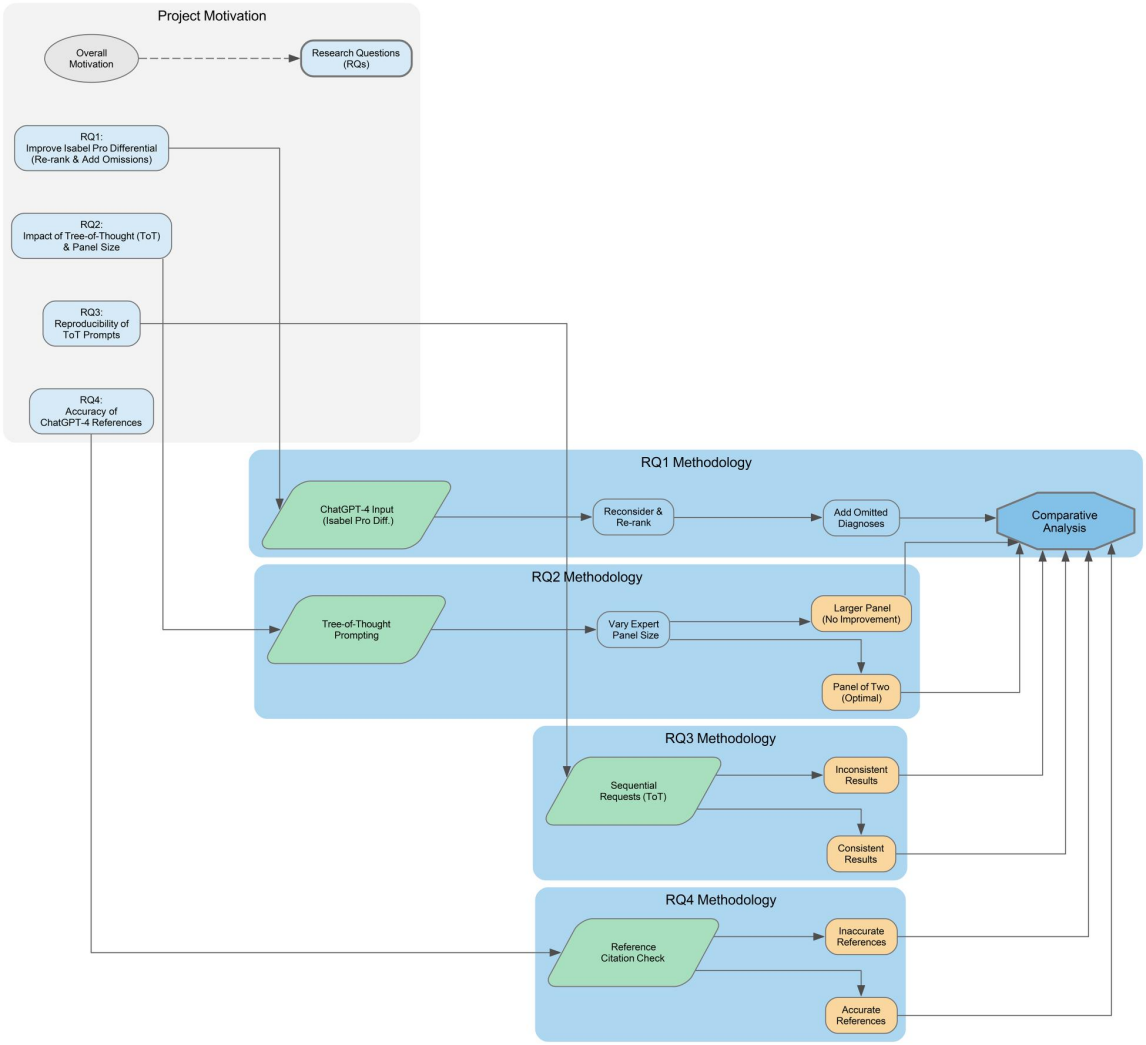
Diagnostic decision support systems: Isabel Pro vs ChatGPT-4.

This study aimed to assess the diagnostic performance of ChatGPT-4 when it was augmented with Isabel Pro’s differential diagnoses. By examining ChatGPT-4’s responses to various prompts—including a Tree-of-Thought approach with an expert panel—this research evaluated improvements in diagnostic accuracy, the consistency of outputs across multiple requests, and the reliability of reference citations. To maintain consistency between previous studies and this study, the same 201 cases were used. The following subsections detail the core research questions, along with the results and discussions derived from the methods described above. Figure 1 shows a high-level overview of our proposed research questions and research strategies.

### Research question 1. Assessing the impact of Isabel Pro’s differential on diagnostic accuracy

#### Does ChatGPT-4 improve diagnostic accuracy when furnished with Isabel Pro’s differential and prompted to reconsider and re-rank the differential? Does allowing the addition of omitted diagnoses enhance diagnostic accuracy?

The first research question contemplates a better performance when asked to reconsider the output of Isabel Pro, reranking the differential presented and adding additional diagnoses if the panel thinks those diagnoses overlooked in the Isabel Pro differential (see Appendix S3). In the next phase of this effort, the prompt entered the entire output of Isabel Pro, followed by a request to review, reconsider, and re-rank the output, but without the option to add diagnoses (see Appendix S4).

### Research question 2. Evaluating the effect of Tree-of-thought and expert panel size

#### What is the effect of a tree-of-thought prompt on the diagnostic accuracy of ChatGPT-4; what is the effect of expert panel size on the diagnostic accuracy of ChatGPT-4?

The second research question used a Tree-of-Thought (ToT) prompt posing a consideration process employing a panel of experts to consider the case inputs, discuss the point of view of each expert, engage in a second consideration, add any diagnoses thought to be missing from the Isabel Pro differential, and produce a consensus differential.^11^ The choice of experts is at the discretion of ChatGPT-4, but one expert must be a Primary Care Physician. (See Appendix S2 for a sample prompt, but the prompt asks ChatGPT-4 to imagine a panel of experts collaborating to produce a differential diagnosis listing. Since this study is designed around initial presentation encounters, one of the experts was required to be a primary care physician). This research question also seeks to determine if the size of the expert panel improves diagnostic accuracy. Each prompt specified the size of the expert panel, ranging from two to ten, with one requesting ChatGPT-4 to choose an “appropriate” panel size.

### Research question 3. Examining consistency across sequential tree-of-thought requests

#### Are the results of Tree-of-Thought prompts to ChatGPT-4 consistent in sequential requests?

A serious question for ChatGPT-4 is whether the differentials produced for a given case are consistent in sequential requests. This question used the 201 Tree-of-Thought prompts for an expert panel size of two to produce five identical requests, comparing the ranking of the correct diagnosis for each case in each of the five requests. The study compared the ranking of the correct diagnosis for each case across each of the five requests to determine whether the results were consistent. For example, if the initial request for a given case produced a rank of one, did each of the following four requests produce a ranking of one, or a different ranking?

### Research question 4. Verifying the accuracy of reference citations

#### What is the accuracy of the reference citations produced by ChatGPT-4 using a Tree-of-Thought prompt?

A frequent criticism of ChatGPT-4 is hallucination or the fabrication of references.^6^ This research question focused on the accuracy of the citations reported. The Tree-of-Thought prompt for an expert panel size of two requested a complete reference citation for each diagnosis presented, including authors, title, journal, page numbers, DOI, and a basis for the ranking likelihood. The authors conducted an internet search for each correct diagnosis to confirm the reference citation.

## Statistical methods

This section outlines the key statistical measures used to evaluate the performance and consistency of the diagnostic approach. We use factors such as mean reciprocal rank, recall at various ranks, and changes in rank under each of the different prompt requests to assess the reliability and accuracy of the model’s outputs. Table 1 explains these metrics, the prompt request comparisons, and their relevance in interpreting the study’s overall results.

**Table 1.** Overview of the key statistical methods and metrics applied in the study.

Method/statistic	Description
Mean reciprocal rank (MRR)	The average of the inverse of the rank for all cases.
Recall at rank	Measures the occurrence of the correct diagnosis within a specified rank. It records a value of 1 if the diagnosis appears at or before that rank and averages this value across all cases.
Average rank and number of correct diagnoses	Average rank: The simple average of the ranks for each of the 201 cases. Number of correct diagnoses: The total count of correct diagnoses identified.
Rank improvement (average, max, min)	For each comparison run, subtract the original rank from the comparison prompt rank. A positive value indicates an improved (higher) rank; a negative value indicates a worse (lower) rank.
Rank change from the first Tree-of-Thought request (average, max, min)	Similar to Rank Improvement, but specifically compares the rank from subsequent runs against the rank in the very first Tree-of-Thought request. The difference is positive if improved and negative if worsened.
Exponential trend line for reproducibility comparison	For each comparison, the study plots the original run’s correct diagnosis rank against the comparison run’s rank in a scatter plot. An exponential trend line fitted to the original run provides an r² (coefficient of determination) value (0 = no fit, 1 = perfect fit).

This study did not test for system equivalence since the Wilcoxon Signed Rank Sum Test from the earlier study^10^ confirmed that the sample size was too small to conclude that the systems are equivalent.

## Results

### Research question one

The Tree-of-Thought prompt expanded in two separate runs for this research question. The first run presented the patient presentations and included the differential produced by Isabel Pro, asking ChatGPT-4 to reconsider and potentially add omitted diagnoses (see Appendix S2 for an example). For this run, the performance statistics improved over the previous runs, with Recall@10 = 0.711, an improvement of 9%. A second run asked for a reconsideration of the Isabel Pro differential without the option to add any diagnoses (see Appendix S3 for an example). This run produced the additional improvement with a Recall@10 = 0.720, an increase of 11%, with the average rank improved by 2.98 and a total of 186 correct diagnoses. Table 2 shows a comparison of these results.

**Table 2.** Comparison of results for research question one on proposed statistics metrics.

	Isabel Pro	Chat GPT4	Tree-of-Thought with option to add diagnoses	Tree-of-Thought, expert panel 2, no added diagnoses
Mean reciprocal rank	0.387	0.436	0.472	0.440
Recall@10	0.650	0.687	0.711	0.720
ChatGPT-4 avg improvement	n/a	−1.478	1.378	2.980
Number of correct diagnoses	175	164	175	186

### Research question two

For this research question, ChatGPT-4 began with the patient’s presentation but prompted to imagine a panel of two experts, one of which was a primary care physician, who would consider the case, discuss their initial differential, and then reconsider and re-rank that differential (see Appendix S1 for an example). This initial Tree-of-Thought prompt did not produce improved results, as shown below, compared to the earlier two attempts.

The prompt required an expert panel size of a specific number, ranging from 2 to 10, with one prompt offering ChatGPT-4 the option to choose an appropriate panel size. For this set of runs, a panel size of two produced the best results, with no panel size greater than two producing an improved result. Table 3 summarizes our comparison.

**Table 3.** Comparison of results for research question 2 on proposed statistics metrics.

	Isabel Pro	Chat GPT-4	T-o-T Prompt	Expert panel size									
				2	3	4	5	6	7	8	9	10	Appropriate
Mean reciprocal rank	0.387	0.436	0.360	0.44	0.41	0.44	0.38	0.42	0.42	0.42	0.40	0.39	0.40
Recall@10	0.650	0.687	0.607	0.72	0.71	0.70	0.66	0.66	0.71	0.69	0.66	0.63	0.66
ChatGPT-4 avg improvement	n/a	−1.478	−4.139	2.98	1.19	2.08	−0.58	0.33	1.66	1.02	−0.39	−0.34	−0.37
Number of correct diagnoses	175	164	156	186	178	184	174	176	183	177	173	175	173

### Research question three

ChatGPT-4 does not produce the same response when subsequently posed with an identical prompt. Repeating the prompt in a series of five sequential runs for a panel size of two demonstrates the degree to which subsequent results differed from the initial run. The aggregate results shown in the table below vary in a narrow range of MRR, Recall@10, and changes in average ranking. However, a valid concern is the sizeable number of better and worse diagnoses, frequently by a wide margin. This lack of reproducibility will make the typical clinician reluctant to use this program for diagnostic assistance.

**Figure 2.**
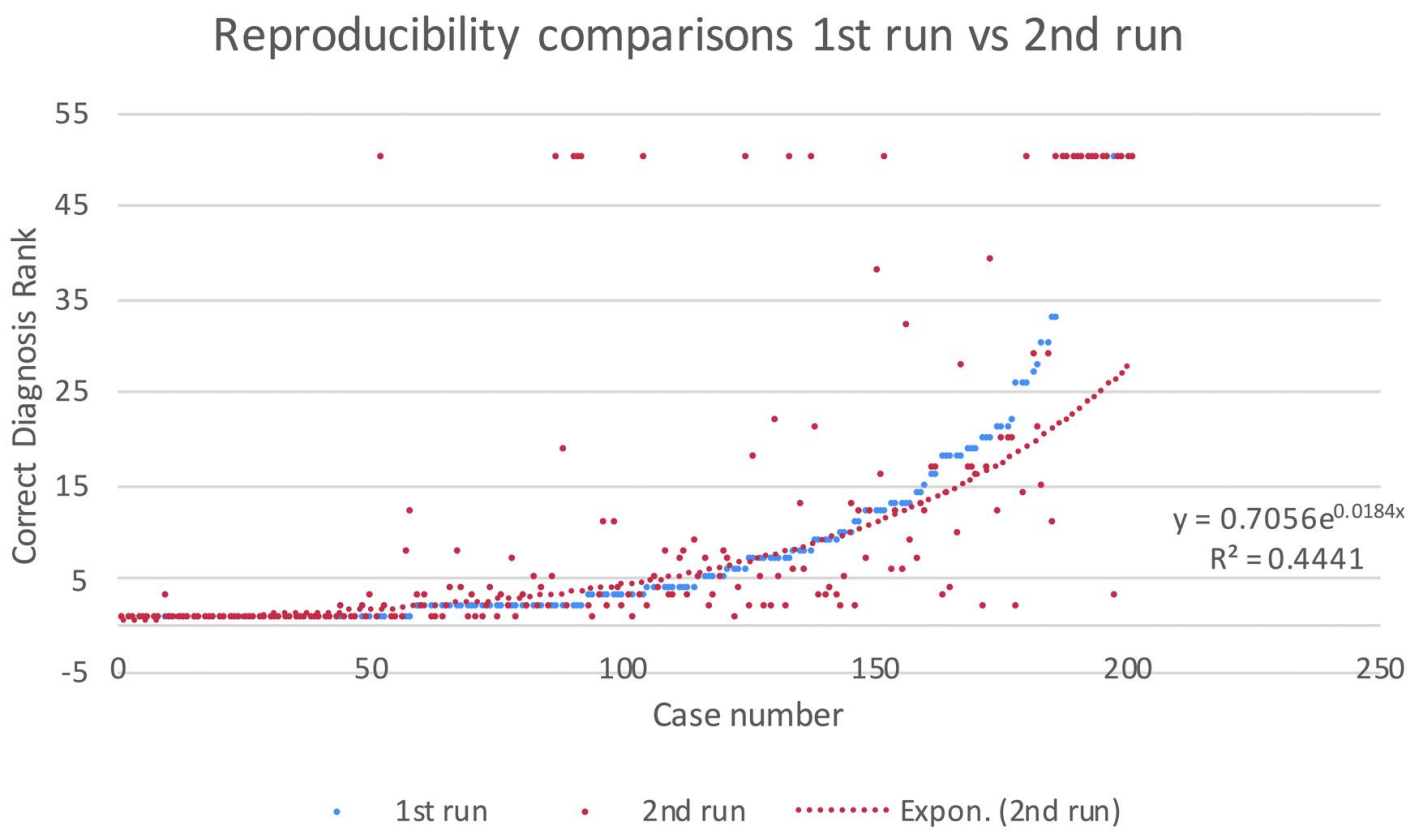
Comparison of correct diagnosis ranks between the first and second runs for each case (blue vs red), with an exponential trend line (red dotted) showing the fit for the second run.

While the initial run with an expert panel size of two achieved a Recall@10 of 0.72, the prompt did not consistently achieve this Recall. Across five runs testing reproducibility, a recall@10 of 0.72, 0.69, 0.711, 0.711, and 0.721 was observed, respectively. With recall@10 averaging approximately 0.71, the outcomes indicate that while not perfectly identical, variance is relatively low, and average results are somewhat consistent. However, when comparing rankings for this series of runs, 63 rankings were better, 56 were worse, and 82 were the same as the first run. The cases were ordered in the sequence of the correct diagnosis rank in Expert Panel 2 and plotted in a scatter plot, comparing each subsequent run with the original run. Figure 2 is an example of the scatter plot. An exponential trend line for the plot of each subsequent run allowed computation of the r-squared, ranging from 0.44 to 0.34, confirming that reproducibility is poor. Table 4 shows the comparison of our results.

**Table 4.** Comparison of results for research question 3 on proposed statistics metrics.

	Expert panel 2	2nd Run	3rd Run	4th Run	5th Run
MRR	0.440	0.439	0.435	0.422	0.438
Recall rank 10	0.720	0.691	0.711	0.711	0.721
Avg improvement over first run	n/a	−1.925	−1.432	−2.164	−1.597
Max rank improvement over first run	n/a	47	48	49	45
Min rank improvement over first run	n/a	−49	−48	−49	−48
Number of better rankings	n/a	64	58	51	63
Number of “same” rankings	n/a	79	82	83	82
Number of worse rankings	n/a	58	61	67	56
r-squared	n/a	0.44	0.34	0.38	0.36

As noted above, the results demonstrate concerns for diagnostic accuracy, reproducibility, and reference citation accuracy. Until these issues are resolved, clinical usage for diagnosis will be minimal, if at all.

### Research question four

Reference accuracy tests showed that citation accuracy and DOI accuracy are very low, with citation accuracy of 34.8% and DOI accuracy of 37.8%. The model produced 70 correct citations and 76 correct DOIs, 91 incorrect citations and 83 incorrect DOIs, and 40 non-existent citations and 42 non-existent DOIs.

### System equivalence

From the earlier study,^10^ the Wilcoxon Signed Rank Sum Test showed that the datasets are too small to allow a definitive determination of system equivalence. For this study, the authors felt it unnecessary to test system equivalence.

### Diagnosis ranking explanation

ChatGPT-4 provides a citation when asked to substantiate the likelihood or ranking of a particular diagnosis but provides little elaboration to support the choice of the cited article or why that particular article supports the diagnosis ranking.

### Reference “hallucination”

As noted in a study by Walters and Wilder^6^ and the earlier study by one of the authors,^10^ ChatGPT-4 demonstrates a disturbing tendency to present incorrect citations. Commonly called “hallucination,” but more realistically “fabrication,” this propensity places the use of ChatGPT-4 in clinical diagnosis as unlikely, or at least with great caution.

## Discussion

No system or combination of systems evaluated in this study accomplished the goal of producing the correct diagnosis within the top ten 95% of the time. In the case of ChatGPT-4, Common Crawl is unlikely to be of adequate reference caliber, and even the most impressive improvement in the model will not overcome the caliber of the training dataset. Providing ChatGPT-4 with a training set composed of the best and most comprehensive medical reference material (for example, the Merck Manual Professional or similar) is likely to be the source of improved diagnostic accuracy. When ChatGPT-4 generates the most likely next word or phrase, the training set becomes not only the source of the diagnosis, but also the source of error since the Common Crawl is composed of all internet medical material, not merely the best medical references. The best performance during this study was the Tree-of-Thought prompt to ChatGPT-4 using an expert panel size of two. That prompt produced a correct diagnosis within the top ten 72% of the time (Recall@10 = 0.72), still well short of Recall@10 = 0.95.

In answer to the first research question, the prompt included the differential produced by Isabel Pro with a request to imagine the panel of experts reconsidering that differential. The prompt offered the option to add any diagnoses. This prompt improved the ranking of the correct diagnosis for the cases, improving Recall@10 from 0.650 to 0.711. The prompt then limited the diagnoses reconsidered and reranked to only those produced by Isabel Pro. This runs further improved Recall@10 to 0.720, improved the average rank by 2.98, and improved the correct cases to 186 from 175.

In the preceding runs, the makeup of the expert panel for each case was determined by ChatGPT-4, leading to the logical research question, “What is the effect of the expert panel on the diagnostic accuracy of ChatGPT-4?” This prompt specified the panel size, ranging from two to ten, and an additional prompt allowed ChatGPT-4 to choose the panel size. An expert panel size of two produced the best results. As it turns out, an expert panel size greater than two hampers the diagnostic accuracy of ChatGPT-4. A reasonable explanation may be the “diagnostic competition” created by more experts.^11^

The second research question introduces the Tree-of-Thought concept of a panel of experts considering the patient's presentation, imagining that the panel will review the patient's presentation, discuss possible differentials, and present a consensus differential. This prompt did not improve diagnostic accuracy. The reason is unclear since ChatGPT-4 offers no thorough explanation for its rankings. This “Black Box” feature of ChatGPT-4 will continue to impede clinicians’ usage.

An essential feature of Isabel Pro is the consistency of its differentials in repeat requests using identical inputs.^8^ A common criticism of ChatGPT-4 is that it produces different answers to repeated requests using identical prompts. This study then prompted ChatGPT-4 in five identical sequential requests. The results were remarkably inconsistent. While the average ranking changed from a high of −1.597 to a low of −2.164, the individual ranking changed from a maximum improvement of +49 to a minimum of −49, with only about 80 diagnoses having the same ranking. A scatter plot of the five runs showed an r-squared ranging from 0.44 to 0.34. Such a wide range of variability demonstrated here is clinically unacceptable and will, until corrected, hinder clinical usage, much like the “Black Box” issue.^14^

The results accompanying research question four demonstrate the magnitude of the “hallucination” or “fabrication” issue with ChatGPT-4, with only about 35% of the references correct, about 45% incorrect, and roughly 20% non-existent.

There are no issues more detrimental to using ChatGPT-4 in medical diagnosis than the lack of reproducibility and the fabrication of references.^6^^,^^15^ At the present time, there are likely no practical strategies for mitigating these issues. The cases in this study come with a known correct diagnosis, but this is not the case in clinical practice. A program that cannot produce a consistent, reproducible differential listing, nor produce a reliable reference for a diagnosis is unlikely to find favor with most, if not all practicing clinicians. Until ChatGPT-4 is validated as a reliable diagnostic tool, the enthusiasm for AI in diagnosis will remain tempered. A future study contemplated by the authors may avoid the fabrication issue since the reference source will be specified and of unquestioned quality. Also, reproducibility might improve when using only information from a high-quality medical reference source.

In addition to a highly respected training dataset, the future of AI in diagnosis also includes the need for smooth integration into the clinical workflow. Integration would also require a faster response method (rather than the current slow typing process). Producing correct citations that can quickly be accessed is another imperative for smooth clinical workflow. Field testing of these improvements with practicing clinicians is essential since physician acceptance is a significant hurdle for diagnostic support.

### Limitations of this study

This study is limited in its generalizability by the size of the dataset. Despite the spread of patient ages, conditions, and medical specialties, the data set is nonetheless small. A format for smooth integration of ChatGPT-4 into the clinical workflow is also unclear. While this study concentrated on diagnostic accuracy and repeatability, the need for a solution to smoothly fit the process into today’s clinical practices will ultimately be an essential element.

## Conclusion

Computerized diagnostic decision support systems have been produced repeatedly for years, beginning as early as the 1970s and continuing until now. Two significant obstacles remain: a smooth interface with the clinical process in practice and diagnostic accuracy sufficient to convince physicians that their use is not only justified but essential. For this study, the desired goal of Recall@10 = 0.95 has been challenging. The highest Recall@10 achieved in this study was 0.720, which required the combination of Isabel Pro and ChatGPT-4 and careful prompt engineering. The results, however, encourage the authors to continue to work toward integrating AI models like ChatGPT-4 into diagnostic processes with training on highly recognized medical reference sources.

Isabel Pro is an advanced computerized diagnostic decision support system that is easy and fast to use but also highly accurate, with a reference database curated over 25 years. Isabel Pro's differentials are reproducible, and the links take the user to the best medical reference sources in seconds. Studies show that Isabel Pro improves the diagnostic accuracy of physicians of all experience levels, whether used early in the diagnostic process or late, producing the fastest result, the largest number of references, and the easiest to use.^8^^,^^16^

ChatGPT-4, introduced in its current form in the spring of 2023, has been received and placed in services faster than any other piece of software. It is presently doing highly regarded service in analyzing images. ChatGPT shows promise in administrative aspects of practice management. Diagnosis, however, has not proven to be as promising. The entry process is cumbersome and time-consuming, but the most concerning issues are diagnostic accuracy, reproducibility, and a clear basis for the diagnosis ranking. Until the resolution of these issues produces absolute accuracy and reproducibility, usage will be minimal and likely limited to administrative tasks, clinical notes, and radiology (where it has demonstrated performance superiority).

Some studies estimate that diagnostic accuracy in the United States may result in as many as 12 million diagnostic errors annually, roughly half resulting in patient harm.^17^ The need to improve diagnostic accuracy should be today's medical imperative. A specific medical reference training dataset (for example, the Merck Manual Professional, the Cochrane Library, or UpToDate) is likely the most critical modification if artificial intelligence is to become accepted in medical diagnosis. Integration modifications for easy inclusion in the clinical workflow are next. An admission process for entering patient data and producing the resulting differential for use by the clinician at the moment of patient encounter is easily as important as an accurate differential listing. As promising as generative, pre-trained, large language models are, this study demonstrates that much remains for artificial intelligence in medical diagnosis to realize its full potential.

## Supplementary Material

ooaf048_Supplementary_Data

## Data Availability

The data underlying this article are available in the New England Journal of Medicine.

## References

[1] Hadi A , Tran E, Nagarajan B, Kirpalani A. Evaluation of ChatGPT as a diagnostic tool for medical learners and clinicians. Ata F, editor. Plos One. 2024;19:e0307383. 39083523 10.1371/journal.pone.0307383 PMC11290643

[2] Cabral S , Restrepo D, Kanjee Z, et al. Clinical reasoning of a generative artificial intelligence model compared with physicians. *JAMA Intern Med* [Internet]. 2024 Apr 1 [cited 2024 Apr 19]. https://jamanetwork.com/journals/jamainternalmedicine/fullarticle/2817046 10.1001/jamainternmed.2024.0295 PMC10985627 38557971

[3] Van Veen D , Van Uden C, Blankemeier L, et al Adapted large language models can outperform medical experts in clinical text summarization. Nat Med. 2024;30:1134-1142. 38413730 10.1038/s41591-024-02855-5 PMC11479659

[4] Thirunavukarasu AJ , Ting DSJ, Elangovan K, Gutierrez L, Tan TF, Ting DSW. Large language models in medicine. Nat Med. 2023;29:1930-1940. 37460753 10.1038/s41591-023-02448-8

[5] Newman-Toker DE , Nassery N, Schaffer AC, et al Burden of serious harms from diagnostic error in the USA. *BMJ Qual Saf* [Internet]. 2023 Jul 17 [cited 2023 Jul 19]. https://qualitysafety.bmj.com/content/early/2023/07/16/bmjqs-2021-014130 10.1136/bmjqs-2021-014130 PMC10792094 37460118

[6] Walters WH , Wilder EI. Fabrication and errors in the bibliographic citations generated by ChatGPT. Sci Rep. 2023;13:14045. 37679503 10.1038/s41598-023-41032-5 PMC10484980

[7] Hirosawa T , Harada Y, Mizuta K, Sakamoto T, Tokumasu K, Shimizu T. Evaluating ChatGPT-4’s accuracy in identifying final diagnoses within differential diagnoses compared with those of physicians: experimental study for diagnostic cases. JMIR Form Res. 2024;8:e59267. 38924784 10.2196/59267 PMC11237772

[8] Riches N , Panagioti M, Alam R, et al The effectiveness of electronic differential diagnoses (DDX) generators: a systematic review and meta-analysis. PloS One. 2016;11:e0148991. 26954234 10.1371/journal.pone.0148991 PMC4782994

[9] McDuff D , Schaekermann M, Tu T, et al Towards accurate differential diagnosis with large language models. *Nature* [Internet]. 2025 Apr 9 [cited 2025 Apr 11]. https://www.nature.com/articles/s41586-025-08869-4 10.1038/s41586-025-08869-4 PMC12158753 40205049

[10] Bridges JM. Computerized diagnostic decision support systems—a comparative performance study of Isabel Pro vs ChatGPT4. 2024; May 7 [cited 2024 May 7] Diagnosis [Internet]. https://www.degruyter.com/document/doi/10.1515/dx-2024-0033/html 10.1515/dx-2024-0033 38709491

[11] Yao S , Yu D, Zhao J, et al Tree of thoughts: deliberate problem solving with large language models [Internet]. arXiv; 2023 [cited 2024 Aug 31]. https://arxiv.org/abs/2305.10601

[12] Friedman CP , Elstein AS, Wolf FM, et al Enhancement of clinicians’ diagnostic reasoning by computer-based consultation: a multisite study of 2 systems. JAMA. 1999;282:1851-1856. 10573277 10.1001/jama.282.19.1851

[13] Wu HW , Davis PK, Bell DS. Advancing clinical decision support using lessons from outside of healthcare: an interdisciplinary systematic review. BMC Med Inform Decis Mak. 2012;12:90. 22900537 10.1186/1472-6947-12-90 PMC3524755

[14] Bathaee Y. The Artificial Intelligence Black Box and the Failure of Intent and Causation. Harv JL & Tech. 2017;31: 889.

[15] Lee P , Goldberg C, Kohane I. The AI Revolution in Medicine: GPT-4 and Beyond. 1st ed. Pearson; 2023.

[16] Sibbald M , Monteiro S, Sherbino J, LoGiudice A, Friedman C, Norman G. Should electronic differential diagnosis support be used early or late in the diagnostic process? A multicentre experimental study of Isabel. *BMJ Qual Saf* [Internet]. 2021 Oct 1 [cited 2021 Oct 11]. https://qualitysafety.bmj.com/content/early/2021/10/04/bmjqs-2021-013493 10.1136/bmjqs-2021-013493 PMC9132870 34611040

[17] Singh H , Meyer AND, Thomas EJ. The frequency of diagnostic errors in outpatient care: estimations from three large observational studies involving US adult populations. BMJ Qual Saf. 2014;23:727-731. 10.1136/bmjqs-2013-002627 PMC4145460 24742777

